# Ginkgetin effectively mitigates collagen and AA‐induced platelet activation via PLCγ2 but not cyclic nucleotide‐dependent pathway in human

**DOI:** 10.1111/jcmm.18139

**Published:** 2024-02-09

**Authors:** Chih‐Wei Hsia, Lan‐Hsin Shu, Ai‐Wei Lee, Oanh‐Thi Tran, Chih‐Hao Yang, Ting‐Lin Yen, Wei‐Chieh Huang, Chih‐Hsuan Hsia, Thanasekaran Jayakumar, Kuan‐Rau Chiou, Joen‐Rong Sheu

**Affiliations:** ^1^ Graduate Institute of Medical Sciences, College of Medicine Taipei Medical University Taipei Taiwan; ^2^ Department of Medical Research Taipei Medical University Hospital Taipei Taiwan; ^3^ Graduate Institute of Pharmacology, College of Medicine National Taiwan University Taipei Taiwan; ^4^ Department of Anatomy and Cell Biology, School of Medicine, College of Medicine Taipei Medical University Taipei Taiwan; ^5^ International Master/Ph.D. Program in Medicine, College of Medicine Taipei Medical University Taipei Taiwan; ^6^ Department of Medical Research Cathay General Hospital Taipei Taiwan; ^7^ Translational Medicine Center Shin Kong Wu Ho‐Su Memorial Hospital Taipei Taiwan; ^8^ Department of Ecology and Environmental Sciences Pondicherry University Puducherry India; ^9^ Division of Cardiology, Department of Internal Medicine, Shuang Ho Hospital Taipei Medical University New Taipei City Taiwan

**Keywords:** ginkgetin, human platelets, MAPK, PI3K/Akt, PLCγ2, vascular thrombosis

## Abstract

Platelets assume a pivotal role in the cardiovascular diseases (CVDs). Thus, targeting platelet activation is imperative for mitigating CVDs. Ginkgetin (GK), from *Ginkgo biloba* L, renowned for its anticancer and neuroprotective properties, remains unexplored concerning its impact on platelet activation, particularly in humans. In this investigation, we delved into the intricate mechanisms through which GK influences human platelets. At low concentrations (0.5–1 μM), GK exhibited robust inhibition of collagen and arachidonic acid (AA)‐induced platelet aggregation. Intriguingly, thrombin and U46619 remained impervious to GK's influence. GK's modulatory effect extended to ATP release, P‐selectin expression, intracellular calcium ([Ca^2+^]i) levels and thromboxane A_2_ formation. It significantly curtailed the activation of various signaling cascades, encompassing phospholipase Cγ2 (PLCγ2)/protein kinase C (PKC), phosphoinositide 3‐kinase/Akt/glycogen synthase kinase‐3β and mitogen‐activated protein kinases. GK's antiplatelet effect was not reversed by SQ22536 (an adenylate cyclase inhibitor) or ODQ (a guanylate cyclase inhibitor), and GK had no effect on the phosphorylation of vasodilator‐stimulated phosphoprotein^Ser157^ or ^Ser239^. Moreover, neither cyclic AMP nor cyclic GMP levels were significantly increased after GK treatment. In mouse studies, GK notably extended occlusion time in mesenteric vessels, while sparing bleeding time. In conclusion, GK's profound impact on platelet activation, achieved through inhibiting PLCγ2–PKC cascade, culminates in the suppression of downstream signaling and, ultimately, the inhibition of platelet aggregation. These findings underscore the promising therapeutic potential of GK in the CVDs.

## 1 INTRODUCTION

Ginkgetin (GK; C_32_H_22_O_10_) is a naturally occurring biflavone isolated from *Ginkgo biloba* L.,^1^ which is one of the most mysterious and oldest Chinese plant species successfully grasp the attention of researchers. The seeds and leaves of *G. biloba* have spiritual and enormous medicinal values in the Chinese culture. GK has been shown to possess several remarkable pharmacological activities. It displays several anticancer properties, such as leukaemia, lung, colon, breast, kidney, prostate, cervical and ovarian cancers through cell cycle arrest, triggering apoptosis, inducing autophagy and preventing angiogenesis.^2^ As far as anti‐inflammation, GK also diminished the expression of inflammation‐associated proteins such as inducible nitric oxide synthase (iNOS), intercellular adhesion molecule 1 (ICAM‐1), cyclooxygenase‐2, prostaglandin E_2_, interleukins and TNF‐α in vitro and in vivo studies.^2^ GK exhibits neuroprotection against neuronal cell damage in Parkinson's disease model by reducing intracellular reactive oxygen species (ROS), sustaining matrix metalloproteinase, suppressing tyrosine hydroxylase in the substantia nigra and increasing superoxide dismutase activity in the striatum.^3^ GK also exerted neuroprotective effects in the brain by decreasing plasma amyloid β and amyloid β plaque levels, inhibiting cerebral microhemorrhage and reducing astrogliosis in APP/PS1 mice.^4^, ^5^ Additionally, GK exhibits neuroprotection against ischemia/reperfusion (I/R)‐induced rat injury by downregulating pro‐inflammatory cytokines and blocking the TLR4/NF‐κB pathway.^6^ The findings highlight the potential clinical application of GK for treating conditions such as Alzheimer's disease and ischemic stroke. For cardiovascular diseases (CVDs), GK exhibited substantial vasodilation via inhibition of cyclic GMP‐phosphodiesterase‐5 and enhanced endothelial NOS expression.^7^, ^8^ In addition, it also significantly diminished atherosclerosis in rat through increasing high‐density lipoprotein while reducing low‐density lipoprotein, and triglyceride levels in blood serum, hence, improved the lipid profile.^8^

Arterial thrombosis stands as the primary cause of cardio/cerebrovascular diseases, such as myocardial infarction, ischemic stroke, venous thromboembolism and peripheral artery diseases, which remain the leading causes of death worldwide. When the connective tissues beneath the vascular endothelium sustain damage, platelets initiate crucial processes of adhesion and aggregation, facilitating the formation of a platelet plug to halt bleeding (haemostasis). While the primary role of platelets is to prevent blood loss following tissue injury, they also play a significant role in the formation of pathogenic blood clots, leading to vascular thromboembolic diseases.

Platelets, anucleated blood cells, are formed from megakaryocytes.^9^ Under normal circumstances, platelets remain inactive. However, the initiation of an intraluminal thrombosis is believed to involve the activation of platelets, including their adherence and aggregation. Platelets cannot aggregate by themselves while circulating normally. Yet, when a blood vessel suffers damage, platelets adhere to the disrupted surface, leading to the release of biologically active substances and subsequent aggregation.^10^ Furthermore, collagen can facilitate platelet adhesion and activation by interacting with collagen receptors, which triggers the release of ADP and the formation of thromboxane A_2_ (TxA_2_).

Antiplatelet drugs are pharmacological agents designed to inhibit excessive activation of platelets, with the purpose of preventing vascular thrombotic diseases. Currently, the most commonly recommended antiplatelet agents include, integrin α_IIb_β_3_ antagonists,^11^ cyclooxygenase inhibitors (i.e. aspirin)^12^ and ADP receptor antagonist.^13^ However, the use of these drugs is associated with an increased risk of bleeding. Therefore, there is a need for further research to develop highly effective and safe agents for inhibition of platelet aggregation in humans.

Several bioactive alkaloids, including ginkgolides, bilobalide and flavonoids, such as GK, have been isolated from *Ginkgo biloba* L. These compounds have demonstrated various noteworthy pharmacological activities.^14^ However, to date, no research has been conducted on the effects of GK specifically on platelet activation, especially its role in human platelets. Therefore, the present study aimed to investigate the antiplatelet effects of GK in humans and elucidate the underlying mechanisms using both ex vivo and in vivo models.

## 2 MATERIALS AND METHODS

### 2.1 Chemicals, reagents and antibodies

GK (>98%), the cyclic AMP, cyclic GMP and thromboxane B_2_ ELISA kits were purchased from Cayman Chemical (Ann Arbor, MI, USA). Collagen (type I), aspirin, bovine serum albumin (BSA), luciferin–luciferase, arachidonic acid (AA), 9,11‐dideoxy‐11α,9α‐epoxymethanoprostaglandin (U46619), 9‐(Tetrahydro‐2‐furanyl)‐9H‐purin‐6‐amine (SQ22536), 1H‐[1,2,4]Oxadiazolo[4,3‐a]quinoxalin‐1‐one (ODQ), heparin, prostaglandin E_1_ (PGE_1_), nitroglycerin (NTG), phenylmethylsulfonyl fluoride (PMSF), sodium orthovanadate, sodium pyrophosphate, aprotinin, leupeptin, sodium fluoride (NaF) and thrombin were purchased from Sigma (St. Louis, MO, USA). Anti‐phospho‐p38 mitogen‐activated protein kinase (MAPK) (Thr^180^/Tyr^182^), anti‐phospho‐Jun N‐terminal kinase (JNK) (Thr^183^/Tyr^185^), anti‐phospholipase C (PLC)γ2, anti‐phospho‐p44/p42 extracellular signal‐regulated kinase (ERK) (Thr^202^/Tyr^204^), anti‐phospho‐phosphoinositide 3‐kinase (PI3K) p85 (Tyr^458^)/p55 (Tyr^199^) and anti‐phospho‐(Ser) protein kinase C (PKC) substrate polyclonal antibodies (pAbs) and anti‐Akt, anti‐p38 MAPK, p44/42 MAPK (ERK1/2; 3A7), anti‐phospholipase Cγ2 (PLCγ2) and anti‐PI3K p85 (19H8) monoclonal antibodies (mAbs) were purchased from Cell Signaling (Beverly, MA, USA). Protein assay dye reagent concentrate was purchased from Bio‐Rad Laboratories Inc. (Hercules, CA, USA). Anti‐phospho‐Akt (Ser^473^) pAb was purchased from BioVision, Inc. (Mountain View, CA, USA). anti‐phospho‐glycogen synthase kinase 3α/β (GSK3α/β), anti‐α‐tubulin and anti‐GSK3α/β mAbs were purchased from Santa Cruz Biotechnology (Santa Cruz, CA, USA). Anti‐vasodilator‐stimulated phosphoprotein (VASP) (phospho Ser^157^), anti‐VASP (phospho Ser^239^) and anti‐VASP pAbs were purchased from Genetex (Irvine, CA, USA). Fura‐2‐acetoxymethyl ester (Fura 2‐AM) was purchased from Molecular Probes (Eugene, OR, USA). FITC‐anti‐human CD42P (P‐selectin) mAb was obtained from BioLegend (San Diego, CA, USA). Amersham (Buckinghamshire, UK) supplied Hybond‐P polyvinylidene difluoride membranes, enhanced chemiluminescence Western blotting detection reagent, horseradish peroxidase‐conjugated donkey anti‐rabbit immunoglobulin G (IgG) and sheep anti‐mouse IgG. A 0.1% dimethyl sulfoxide (DMSO) was used to dissolve GK and the stock solution was stored at 4°C.

### 2.2 Preparation of washed human platelets and cytotoxic assessment

This study was approved by the Institutional Review Board of Taipei Medical University (TMU‐JIRB‐N202112047) and conducted in accordance with the principles outlined in the Helsinki Declaration. All human blood donors participating in the study provided informed consent by signing a consent form before enrollment. Platelet suspensions were prepared from healthy human blood donors by combining whole blood with an acid‐citrate‐dextrose solution (at a ratio of 9:1, v/v), following a previously described method.^15^ Subsequently, centrifugation at 120 × *g*, 10 min was performed to obtain platelet‐rich plasma (PRP), which was supplemented with EDTA (2 mM) and heparin (6.4 U/mL). After a 5‐min incubation period, another round of centrifugation was conducted at 500 × *g*, 10 min. The resulting platelet pellets were resuspended and centrifuged again, ultimately being suspended in Tyrode's solution (pH 7.3; 137 mM NaCl, 2.4 mM KCl, 1 mM MgCl_2_, 0.2 mM Na_2_HPO_4_, 12 mM NaHCO_3_, 5.5 mM glucose and 3.5 mg/mL BSA) containing Ca^2+^ at 1 mM. Platelet counts were determined using a Coulter counter (Beckman Coulter, Miami, FL, United States). Washed platelets, adjusted to a concentration of 3.6 × 10^8^ cells/mL, were preincubated with a solvent control (0.1% DMSO) or GK (ranging from 0.25 to 100 μM) for a duration of 3 min before being stimulated with various agonists, including collagen (1 μg/mL), AA (60 μM), thrombin (0.02 U/mL) and U46619 (1 μM). The aggregation capacity was assessed using a lumi‐aggregometer (Payton, Scarborough, Ontario, Canada).^15^ The extent of platelet aggregation was quantified as the percentage of aggregation observed in the control group (treated with 0.1% DMSO) based on light transmission units. In the ATP release assay, the platelet suspension was supplemented with luciferin‐luciferase reagent 1 min prior to the addition of collagen. Following this, absorbance measurements were performed using a Hitachi Spectrometer F‐7000 (Tokyo, Japan) to quantitatively analyse the released ATP levels. Furthermore, washed platelets (3.6 × 10^8^ cells/mL) were preincubated with GK (0.5–100 μM) or 0.1% DMSO for 20 min at 37°C. An aliquot of the supernatant (10 μL) was then deposited onto a Fuji Dri‐Chem slide (LDH‐PIII; Fuji, Tokyo, Japan), and absorbance was measured at a wavelength of 540 nm using an ultraviolet–visible spectrophotometer (UV‐160; Shimadzu, Kyoto, Japan). The maximum level of lactate dehydrogenase (LDH) activity was observed in triton‐treated platelets.

### 2.3 Assessment of intracellular calcium level

To assess intracellular calcium level ([Ca^2+^]i), whole blood treated with citrate was centrifuged, and the resulting supernatant was incubated with 0.1% DMSO or GK (0.5 and 1 μM) and Fura 2‐AM (5 μM). The levels of Fura 2‐AM were measured using a Hitachi Spectrometer F‐7000 (Tokyo, Japan) with excitation wavelengths of 340 nm and 380 nm, and an emission wavelength of 500 nm.^16^

### 2.4 Analysis of surface P‐selectin expression

Platelets were treated with GK (0.5 and 1 μM) in combination with FITC‐conjugated anti‐P‐selectin mAb (2 μg/mL). This preincubation step lasted for 3 min. Following the preincubation, the platelets were stimulated with collagen (1 μg/mL). To analyse the platelets, a flow cytometer (FAC Scan system; Becton Dickinson, San Jose, CA, USA) was used to detect fluorescein‐labelled platelets. Data were collected from 50,000 platelets per experimental group, and the platelets were identified based on their characteristic forward and orthogonal light‐scattering profiles.^16^

### 2.5 Measurement of TxB_2_ formation

Platelet suspensions (3.6 × 10^8^ cells/mL) were preincubated with 0.1% DMSO or GK (1 and 5 μM) for 3 min, followed by the addition of collagen (1 μg/mL) or AA (60 μM). 6 min after the addition of agonists, EDTA (2 mM) and indomethacin (50 μM) were added to the suspensions and centrifuged at 2000 × *g* for 5 min. Finally, the TxB_2_ levels were measured from the supernatants using an ELISA kit according to the manufacturer's instructions.

### 2.6 Immunoblotting

Washed platelets (1.2 × 10^9^ cells/mL) were subjected to incubation with GK (0.5 and 1 μM), PGE_1_ (20 nM), NTG (10 μM) or 0.1% DMSO. Following this, the platelets were stimulated with or without agonists for a duration of 5 min. To prepare the platelets for further analysis, a 200 μL lysis buffer (pH 7.4; 46 mM HEPES, 5 mM EDTA.2Na, 50 mM NaCl and 16 μM TritonX‐100) consisting of aprotinin (10 μg/mL), PMSF (1 mM), leupeptin (2 μg/mL), NaF (10 mM), sodium orthovanadate (1 mM) and sodium pyrophosphate (5 mM) was added. The prepared platelets were resuspended in the lysis buffer and incubated for 1 h. After centrifugation at 5000 × *g* for 5 min, the supernatant containing the lysates was collected. From these lysates, 80 μg of protein was separated using 12% SDS‐PAGE. Protein concentrations were determined using the Bradford protein assay (Bio‐Rad, Hercules, CA, USA). To identify specific target proteins, respective primary antibodies were utilized for protein spot detection. The optical density of the protein bands was quantified using a video densitometer and Bio‐profil Biolight software, Version V2000.01 (Vilber Lourmat, Marne‐la‐Vallée, France). The relative protein expression was determined by normalizing the expression to the total protein of interest.

### 2.7 Confocal laser fluorescence microscopy

Resting or collagen‐activated platelets were immobilized on poly‐L‐lysine‐coated coverslips and fixed in a solution containing 4% (v/v) paraformaldehyde for 1 h. Following fixation, platelets were permeabilized using 0.1% triton X‐100 and then incubated in a solution of 5% BSA in phosphate‐buffered saline (PBS) for 1 h to block nonspecific binding sites. Subsequently, platelets were subjected to immunostaining by incubating them with specific primary antibodies targeting the proteins of interest for 24 h. After thorough washing with PBS, the platelets were incubated with secondary antibodies (Alexa Fluor® 488 labelled goat anti‐rabbit IgG and Alexa Fluor® 647 labelled goat‐anti‐mouse IgG) for 1 h. Finally, the platelets were imaged under a confocal microscope (Leica TCS SP5, Mannheim, Germany) equipped with a 100× oil immersion objective. The intensities of immunoreaction were quantified using the NIH ImageJ software program (NIH, Bethesda, MD; http://rsbweb.nih.gov/ij/).

### 2.8 Measurement of cyclic nucleotide formation

Platelet suspensions (3.6 × 10^8^ cells/mL) were incubated with PGE_1_ (20 nM) or NTG (10 μM) or GK (0.5 and 1 μM) in the presence of 100 μM 3‐isobutyl‐1‐methylxanthine for 6 min. Incubation was stopped, and the solution was immediately boiled for 5 min. The supernatants (50 μL) were employed in determining the contents of cyclic AMP and cyclic GMP by using EIA kits.

### 2.9 Microvascular thrombosis in mouse mesenteric vessels by sodium fluorescein irradiation

The experimental procedures and protocols adhered to the guidelines established in the Affidavit of Approval of Animal Use Protocol, as authorized by Taipei Medical University (LAC‐2021‐0386) in Taiwan. In summary, male ICR mice aged 6 weeks were subjected to intraperitoneal injections of either 50 μL of a 0.1% DMSO solution or varying doses of GK at 1 and 2 mg/kg. Following this, intravenous administration of sodium fluorescein at a dose of 15 μg/kg was conducted, employing a methodology previously detailed in another study.^17^ To facilitate the experiments, the ICR mice were anaesthetised via intraperitoneal administration of sodium pentobarbital at a dosage of 50 mg/kg. Venules measuring 30–40 μm in diameter were subjected to irradiation using light with a wavelength below 520 nm to induce the formation of microthrombi. We meticulously recorded the time it took for these thrombi to fully occlude the microvessel, a parameter referred to as ‘occlusion time’.

### 2.10 Evaluation of tail bleeding time in mice

Bleeding time was assessed in male ICR mice using the tail‐vein transection model. Prior to the procedure, mice were sedated with intraperitoneal sodium pentobarbital (50 mg/kg) and intraperitoneally given doses of 1 and 2 mg/kg GK, 0.1% DMSO or 2 mg/kg aspirin (all in 50 μL) for a 30‐min duration. Subsequently, the tails were subjected to transection and immediately immersed in normal saline at 37°C. The duration of bleeding was measured until complete cessation of bleeding occurred.

### 2.11 Statistical analysis

The data are presented as the mean ± standard error of the mean. The value of *n* represents the number of experiments conducted using samples from distinct blood donors. To examine significant differences among the experimental groups, a one‐way analysis of variance (ANOVA) was employed, followed by the Student–Newman–Keuls post hoc test to control for family‐wise type I error. Statistical significance was defined as *p* < 0.05. All statistical analyses were performed using SAS (version 9.2; SAS Inc., Cary, NC, USA).

## 3 RESULTS

### 3.1 GK demonstrates potent efficacy in suppressing platelet aggregation induced by collagen and AA in human platelets

**FIGURE 1:**
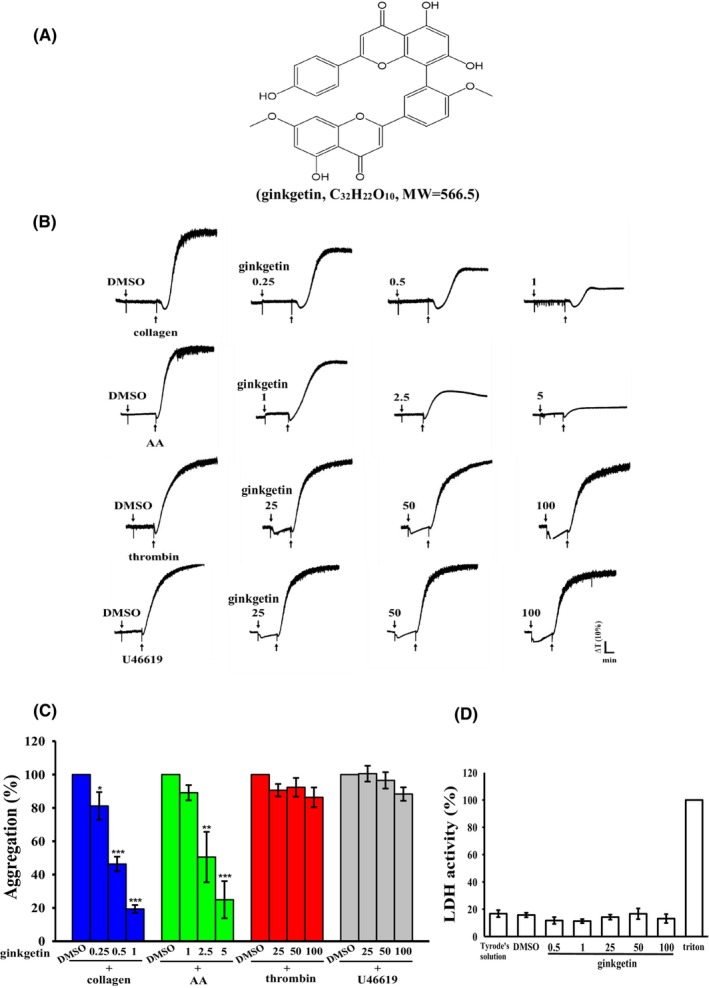
Modulation of platelet aggregation and cytotoxicity by ginkgetin in response to diverse agonists. (A) The chemical structure of ginkgetin, with its molecular formula C_32_H_22_O_10_, is depicted. (B) Washed human platelets, at a concentration of 3.6 × 10^8^ cells/mL, underwent preincubation with either a solvent control (0.1% DMSO) or varying concentrations of ginkgetin (ranging from 0.25 to 100 μM). Subsequently, platelets were exposed to various agonists, including collagen (1 μg/mL), arachidonic acid (AA; 60 μM), thrombin (0.02 U/mL) or U46619 (1 μM), in order to incite platelet aggregation. (C) Concentration‐response curves for ginkgetin reveal its inhibitory potential on platelet aggregation triggered by various agonists (%). (D) To assess cytotoxicity, platelets were pretreated with either a solvent control (0.1% DMSO) or ginkgetin at concentrations ranging from 0.5 to 100 μM for a duration of 20 min. Subsequently, a 10‐μL aliquot of the resulting supernatant was deposited onto a Fuji Dri‐Chem slide LDH‐PIII. Significance levels of *p < 0.05, **p < 0.01 and ***p < 0.001 were employed to denote statistical differences compared to the 0.1% DMSO‐treated group. The data presented in both (C) and (D) as the mean ± standard error of the mean (*n* = 4).

GK (Figure 1A) exhibited remarkable potency in inhibiting platelet aggregation induced by collagen (1 μg/mL) at concentrations ranging from 0.25 to 1 μM. It displayed moderate‐to‐high activity (1–5 μM) by AA (60 μM) stimulation. However, GK had no significant effects by stimulating either thrombin (0.02 U/mL) or U46619 (1 μM), a prostaglandin endoperoxide, even at concentrations as high as 100 μM (Figure 1B,C). The precise IC_50_ values for GK in the inhibition of collagen and AA‐induced platelet activation were determined to be 0.55 and 3.2 μM, respectively. To facilitate experimental procedures and ensure consistent sampling, we opted to standardize the IC_50_ value to 0.5 μM for subsequent investigations into the inhibitory mechanism of GK induced by collagen. Additionally, we conducted another study to further examine the cytotoxicity of GK in platelets. The results revealed that the aggregation curves of platelets preincubated with 50 μM GK for 10 min and subsequently washed twice with Tyrode's solution were not significantly different from those of platelets preincubated with the solvent control (0.1% DMSO) under equivalent conditions (Figure S1). Furthermore, the LDH assay demonstrated that treatment with GK (100 μM) did not induce any notable release of LDH when platelets were pretreated for 20 min (Figure 1D). These findings suggest that the effects of GK on platelet aggregation are reversible and noncytotoxic.

### 3.2 Regulatory insights of GK on ATP release, relative [Ca^2+^]i level and surface P‐selectin expression as well as TxB_2_ formation

**FIGURE 2:**
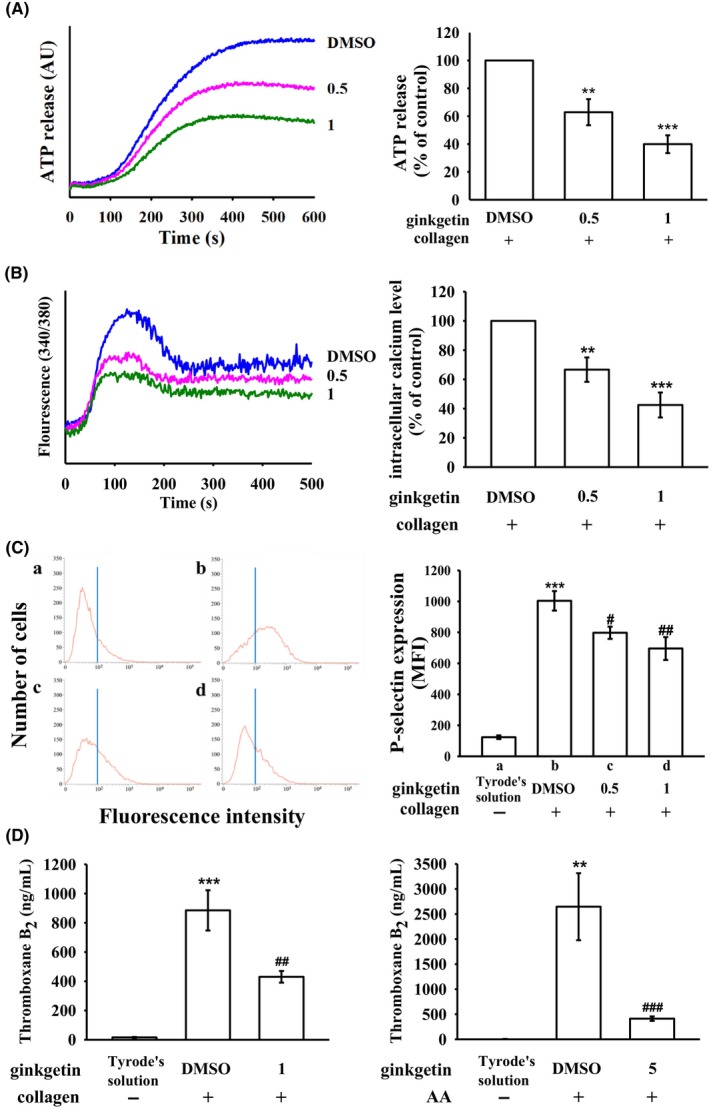
Inhibitory impact of ginkgetin on ATP release, relative [Ca^2+^]i level and surface P‐selectin expression as well as thromboxane B_2_ formation in human platelets. Washed platelets (3.6 × 10^8^ cells/mL) were subjected to preincubation with either 0.1% DMSO or ginkgetin (0.5 and 1 μM), followed by the addition of collagen (1 μg/mL) to instigate the following responses: (A) ATP release, quantified in arbitrary units (AU); (B) relative [Ca^2+^]i level; and (C) surface P‐selectin expression (a. Tyrode's solution, b. collagen‐stimulated platelets, c. 0.5 μM ginkgetin and d. 1 μM ginkgetin); (D) thromboxane B_2_ formation. A comprehensive account of the experimental methodologies employed can be found in the Section 2. The corresponding statistical findings are displayed in the right‐hand panel of each figure. In figure (A), (B), significance levels of **p < 0.01 and ***p < 0.001 are employed to signify distinctions in comparison to the group treated with 0.1% DMSO. In figure (C) and (D), **p < 0.01 and ***p < 0.001 is utilized to denote deviations in comparison to the resting control (Tyrode's solution), while ^#^ *p* < 0.05, ^##^ *p* < 0.01 and ^###^p < 0.001 are employed to indicate differences in comparison to the 0.1% DMSO‐treated group. The data presented as the mean ± standard error of the mean (*n* = 4).

GK, at concentrations of 0.5 and 1 μM, demonstrated a concentration‐dependent reduction in collagen‐induced ATP release (Figure 2A). Additionally, both concentrations of GK significantly attenuated the elevation of intracellular calcium levels ([Ca^2+^]i) induced by collagen, resulting in reductions of approximately 34% and 58%, respectively (Figure 2B). P‐selectin serves as a crucial biomarker for platelet activation. Normally, P‐selectin is localized within the inner walls of α‐granules; however, upon activation, platelets expose the inner granule contents to the outer membrane.^18^ Figure 2C illustrates the suppressive effect of GK (0.5 and 1 μM) on collagen‐stimulated surface FITC‐P‐selectin expression (a, Tyrode's solution, 123.3 ± 11.8; b, 0.1% DMSO + collagen group, 1004.0 ± 62.9; c, 0.5 μM GK + collagen group, 797.8 ± 39.4; d, 1 μM GK + collagen group, 695.8 ± 73.7; *n* = 4). The corresponding statistical data are provided in the right‐hand panels of Figure 2. In Figure 2D, resting platelets produced relatively little TxB_2_ (16 ± 3 ng/mL and 6 ± 1 ng/mL; *n* = 4) compared with collagen‐ (885 ± 138 ng/mL; *n* = 4) or AA‐ (2646 ± 668 ng/mL; *n* = 4) stimulated platelets, respectively. GK, at concentrations of 1 and 5 μM, markedly reduced TxB_2_ formation stimulated by collagen (430 ± 40 ng/mL; *n* = 4) and AA (413 ± 44 ng/mL; *n* = 4), respectively.

### 3.3 Suppression of PLCγ2‐PKC activation by GK

**FIGURE 3:**
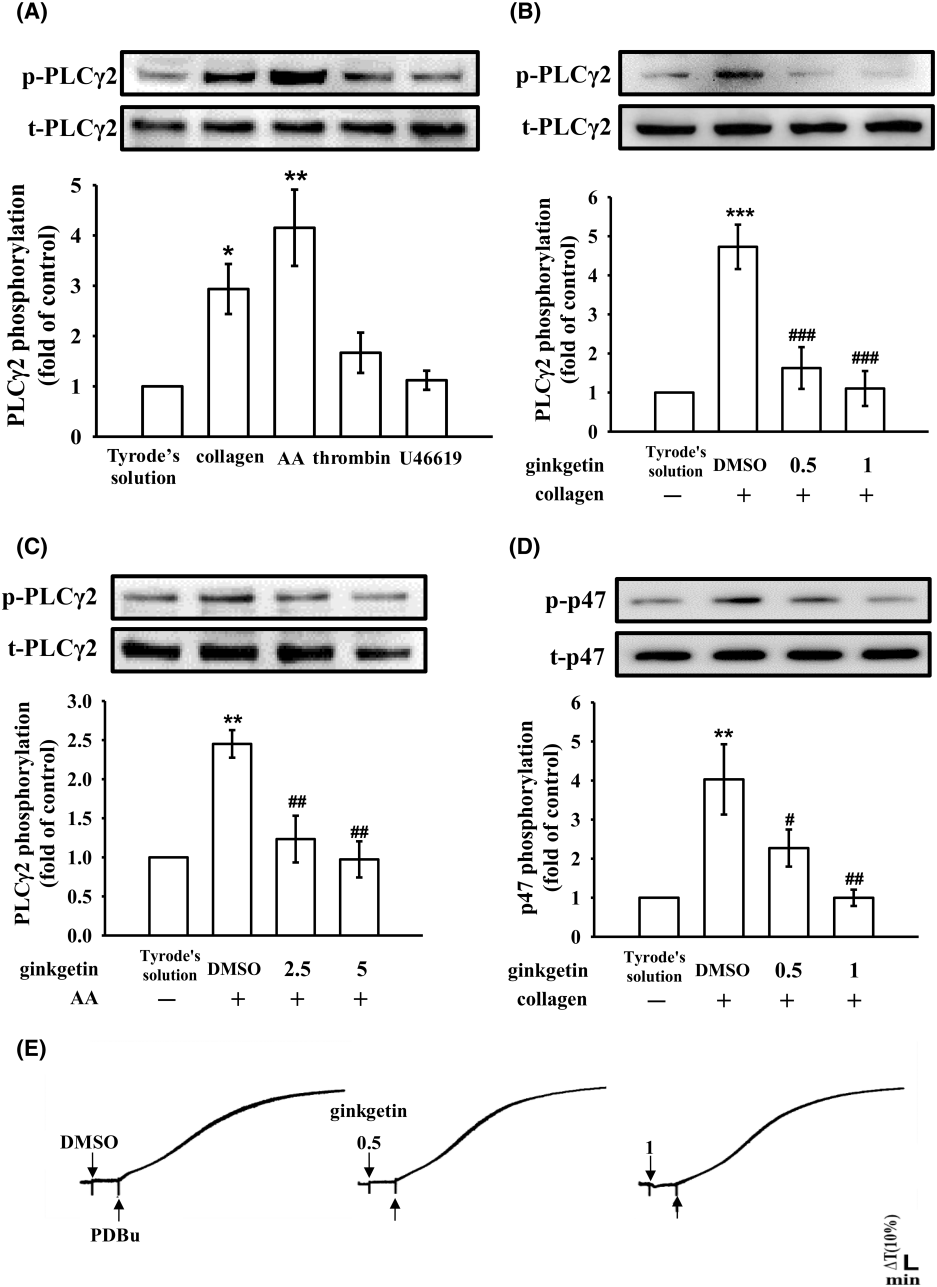
Effects of ginkgetin on the activation of phospholipase Cγ2 (PLCγ2) and protein kinase C (PKC) in platelets. Washed platelets were subjected to preincubation with either 0.1% DMSO or ginkgetin (at concentrations ranging from 0.5 to 5 μM) followed by stimulation with collagen (1 μg/mL), arachidonic acid (AA; 60 μM), thrombin (0.02 U/mL), U46619 (1 μM) or phorbol 12,13‐dibutyrate (PDBu, 150 nM, a PKC activator) to elicit the following responses: (A–C) activation of PLCγ2, (D) activation of PKC as indicated by p‐p47 phosphorylation and (E) the ensuing platelet aggregation. (A–D) Data are presented as the mean ± standard error of the mean (*n* = 4). Significant differences are represented by *p < 0.05, **p < 0.01 and **p < 0.001 in comparison to resting platelets exposed to Tyrode's solution. Furthermore, ^#^ *p* < 0.05, ^##^ *p* < 0.01 and ^###^ *p* < 0.001 are used to denote disparities in comparison to the group treated with 0.1% DMSO. The data in (E) are representative of four independent experiments.

**FIGURE 4:**
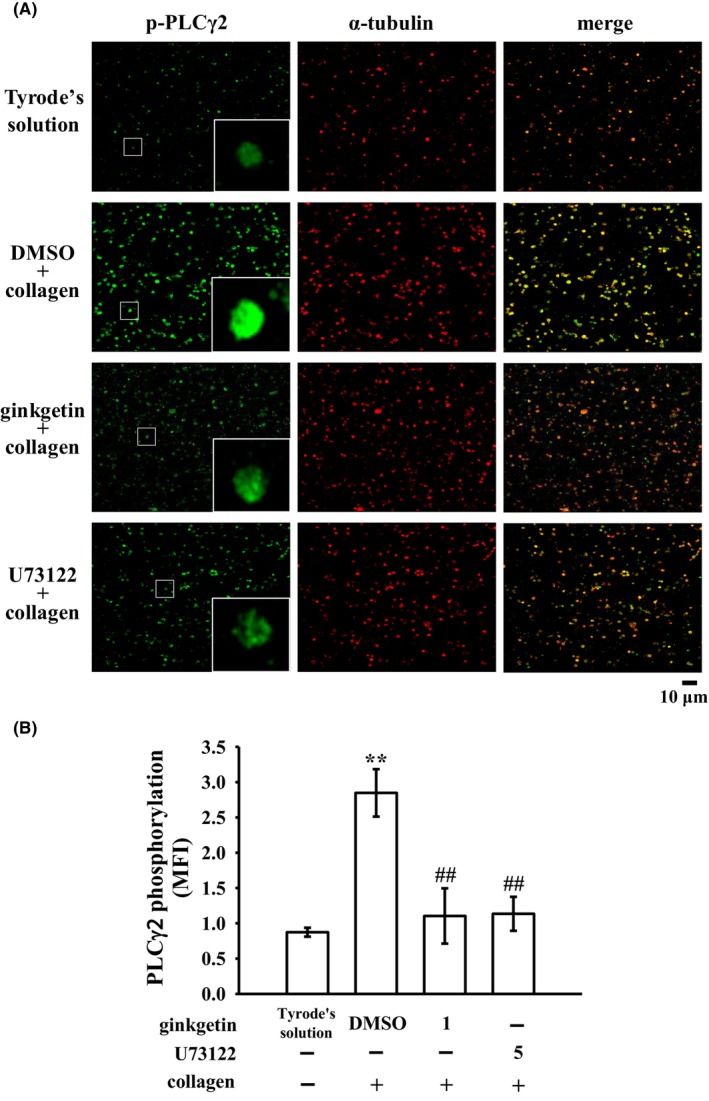
Inhibitory effects of ginkgetin and U73122 on phospholipase Cγ2 (PLCγ2) activation visualized by confocal laser microscopy. Washed platelets were preincubated with 0.1% DMSO, ginkgetin (1 μM) or U73122 (5 μM, a PLC inhibitor) and subsequently exposed to collagen (1 μg/mL) for confocal microscopic evaluation at 1000× magnification. This assessment focused on (A) the visualization of phosphorylated PLCγ2 through green fluorescence and α‐tubulin through red fluorescence. Alexa Fluor® 488 labelled goat anti‐rabbit IgG and Alexa Fluor® 647 labelled goat‐anti‐mouse IgG were employed for this purpose. The (B) contains the associated statistical analysis (MFI: mean of fluorescence intensity). Data are presented as the mean ± standard error of the mean (*n* = 4). Significant differences are indicated by **p < 0.01 in comparison to resting platelets exposed to Tyrode's solution and ^##^ *p* < 0.01 in comparison to the 0.1% DMSO group. Bar: 10 μm.

PLC, a member of the kinase family, catalyses the hydrolysis of phosphatidylinositol 4,5‐bisphosphate, resulting in the production of diacylglycerol (DAG) and inositol 1,4,5‐trisphosphate (IP_3_), which serve as crucial secondary messengers. DAG activates PKC, leading to the phosphorylation of a predominantly 47 kDa protein called pleckstrin (p47) and subsequent ATP release. IP_3_, on the contrary, triggers calcium mobilization from intracellular stock.^19^ Experimental results, as depicted in Figure 3A, clearly indicate that PLCγ2 phosphorylation is prominently induced by collagen and AA, but a weaker response by thrombin and no response by U46619. Furthermore, treatment with GK (0.5–5 μM) markedly attenuated PLCγ2 phosphorylation triggered by collagen and AA (Figure 3B,C). However, we also assessed the effect of GK on thrombin‐induced PLCβ. The result indicated that the phosphorylation of PLCβ was not inhibited by GK (Figure S2). Moreover, we also analysed the impact of GK on IP_3_ levels in collagen‐stimulated platelet activation. The results showed that GK (0.5 and 1 μM) significantly reduces IP_3_ levels, leading to approximately 40% and 64% reductions, respectively (Figure S3). These findings underscore the substantial inhibitory effect of GK on both PLCγ2 phosphorylation and activity. Moreover, GK (0.5 and 1 μM) also obviously diminished PKC activation (pleckstrin; p‐p47) triggered by either collagen (Figure 3D) or AA (data not shown). It should be noted that the antibody used in this immunoblotting recognizes all substrates of PKC, not only p‐p47. However, in platelets, p‐p47 is the major dominant substrate of PKC on platelet activation.^20^ GK (0.5 and 1 μM) did not exhibit a significant reduction in platelet aggregation induced by PDBu (a PKC activator) (Figure 3E). The findings suggest that GK does not directly contribute to PKC activation but likely exerts its effects through the modulation of PKC's upstream regulator, PLCγ2, upon stimulation by collagen and AA. The corresponding statistical data are provided in the lower panels of individual figures. In addition, the inhibitory effect of GK on PLCγ2 phosphorylation was also validated using confocal laser fluorescence microscopy to compare the levels of phosphorylated PLCγ2 (green fluorescence) with that of α‐tubulin (red fluorescence) in resting platelets and collagen‐activated platelets. We observed an increase in the fluorescence intensity of phosphorylated PLCγ2 following stimulation with collagen, compared to the resting group (treated only with Tyrode's solution) (Figure 4A). However, pretreatment with GK (1 μM) or U73122 (a PLC inhibitor; 5 μM) significantly attenuated the enhanced fluorescence intensity of phosphorylated PLCγ2 stimulated by collagen (Figure 4A). The corresponding statistical analysis is provided in the Figure 4B.

### 3.4 Regulatory effects of GK on PI3K‐Akt‐GSK3β and MAPKs activation

**FIGURE 5:**
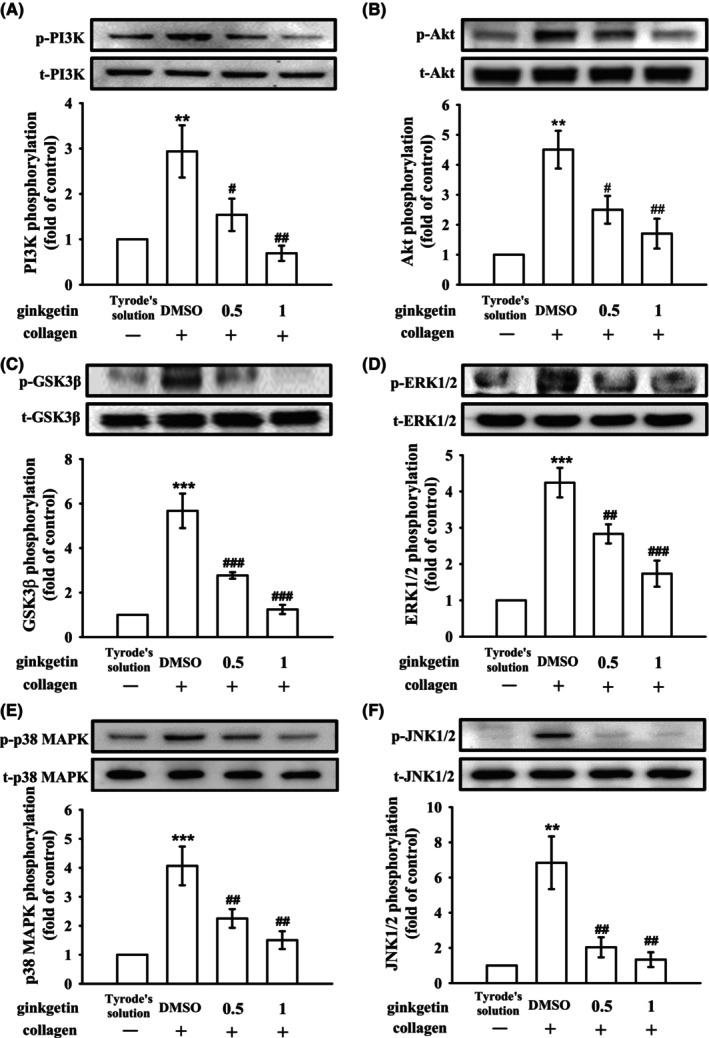
Regulatory influence of ginkgetin on platelet signaling pathways: phosphoinositide 3‐kinase (PI3K)/Akt/glycogen synthase kinase‐3β (GSK3β) and mitogen‐activated protein kinases (MAPKs). Washed platelets were subjected to preincubation with either 0.1% DMSO or ginkgetin (0.5 and 1 μM) and subsequently exposed to collagen (1 μg/mL). This facilitated the immunoblotting analysis of key components within the (A) PI3K, (B) Akt, (C) GSK3β, (D) ERK1/2, (E) p38 MAPK and (F) JNK1/2 pathways. Data are presented as the mean ± standard error of the mean (*n* = 4). **p < 0.01 and ***p < 0.001 compared with the results noted in resting platelets (Tyrode's solution); ^#^ *p* < 0.05, ^##^ *p* < 0.01 and ^###^ *p* < 0.001 compared with the results noted in the 0.1% DMSO group.

The PI3K/Akt/GSK3β signaling pathway has been implicated in thrombus formation under conditions of high shear stress.^21^ PI3K plays a significant role in activating Akt, which serves as the primary regulator in this pathway.^21^ Various platelet agonists that regulate platelet activation and haemostasis can activate the Akt pathway. Additionally, GSK3β is known to be regulated by the PI3K/Akt pathway in platelets.^22^ In our study, GK at concentrations of 0.5 and 1 μM effectively suppressed the activation of the PI3K/Akt/GSK3β pathway in platelets stimulated with collagen (Figure 5A–C). Furthermore, MAPK signaling pathways, including ERK1/2, p38 MAPK and JNK1/2, are known to regulate inflammation, cell proliferation, apoptosis and platelet activation.^23^ Notably, GK (0.5 and 1 μM) attenuated the phosphorylation of all three MAPKs stimulated by collagen, indicating that GK‐mediated antiplatelet activation involves the modulation of MAPK pathways (Figure 5D–F).

### 3.5 Exploring the involvement of intracellular cyclic nucleotides in the antiplatelet activity of GK

**FIGURE 6:**
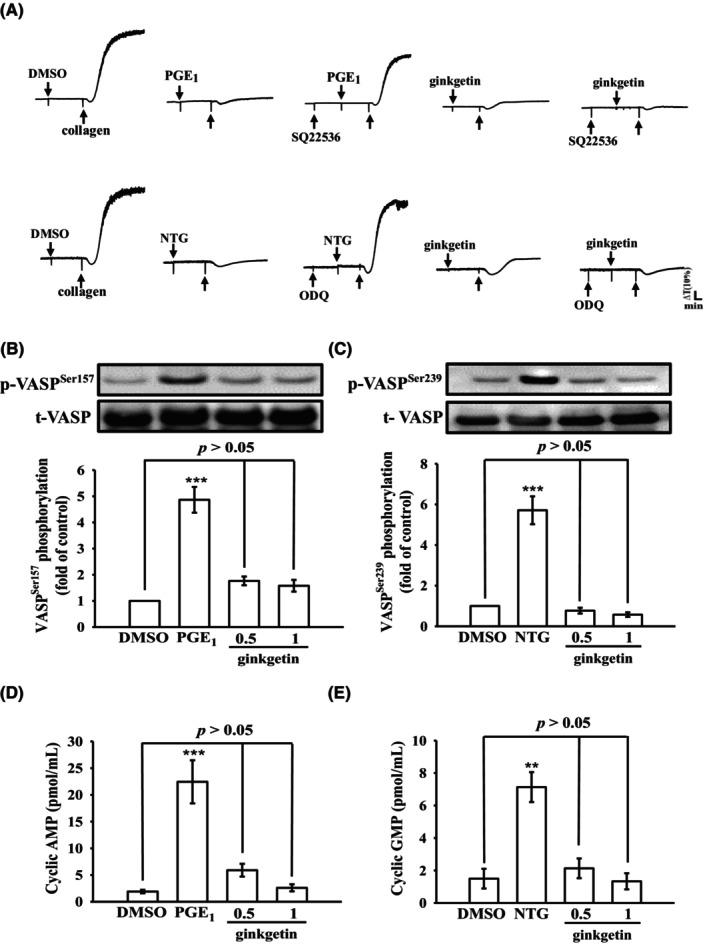
Impact of ginkgetin on cyclic nucleotides and vasodilator‐stimulated phosphoprotein (VASP) phosphorylation as well as cyclic nucleotide formation in human platelets. (A) Washed platelets were preincubated with prostaglandin E_1_ (PGE_1_; 20 nM), nitroglycerin (NTG; 10 μM) or ginkgetin (1 μM), all in the presence of SQ22536 (100 μM, an adenylate cyclase inhibitor) or ODQ (10 μM, a guanylate cyclase inhibitor) for a duration of 3 min prior to the addition of collagen (1 μg/mL) to initiate platelet aggregation. (B–E) Washed platelets were directly stimulated with PGE_1_ (20 nM), NTG (10 μM) or ginkgetin (0.5 and 1 μM) to assess VASP phosphorylation and the levels of cyclic AMP and cyclic GMP, respectively. Profiles in (A) are representative of four independent experiments. All data (B–E) is expressed as the mean ± standard error of the mean (*n* = 4). Significance levels are denoted by **p < 0.01 and ***p < 0.001 when comparing to the 0.1% DMSO group.

Cyclic nucleotides, including cyclic adenosine monophosphate (cyclic AMP) and cyclic guanosine monophosphate (cyclic GMP), are small cyclized monophosphates that serve as crucial second messengers in various signal transduction pathways. These pathways play a vital role in regulating multiple targets, including protein kinases involved in the phosphorylation of VASP. In Figure 6A, we observe the notable inhibitory effects of PGE_1_ (20 nM) and NTG (10 μM), on collagen‐induced platelet aggregation. These inhibitory actions are intriguingly demonstrated to be reversible when exposed to specific inhibitors: SQ22536 (100 μM), known as an adenylate cyclase inhibitor, effectively counters the impact of PGE_1_, while ODQ (10 μM) recognized as a guanylate cyclase inhibitor, similarly reverses the effects of NTG. However, neither SQ22536 nor ODQ significantly reversed the antiplatelet activity mediated by GK (1 μM). Furthermore, while PGE_1_ (20 nM) and NTG (10 μM) prominently stimulated the phosphorylation of VASP^Ser157^ and VASP^Ser239^, respectively, GK (0.5 and 1 μM) had no effect on the phosphorylation of either VASP^Ser157^ or VASP^Ser239^ (Figure 6B,C). Moreover, levels of cyclic AMP and cyclic GMP in resting platelets were lower compared with those of 20 nM PGE_1_‐ and 10 μM NTG‐treated platelets (1.9 ± 0.3 vs. 22.4 ± 4.0 pmol/mL, *n* = 4, Figure 6D; 1.5 ± 0.6 vs. 7.1 ± 0.9 pmol/mL *n* = 4, Figure 6E), respectively. Neither cyclic AMP nor cyclic GMP level was significantly increased after GK treatment (0.5 and 1 μM) (Figure 6D,E). These findings suggest that GK‐mediated inhibition of platelet aggregation does not involve an increase in intracellular cyclic nucleotide formation.

### 3.6 Effectiveness of the antithrombotic activity of GK in animal studies

**FIGURE 7:**
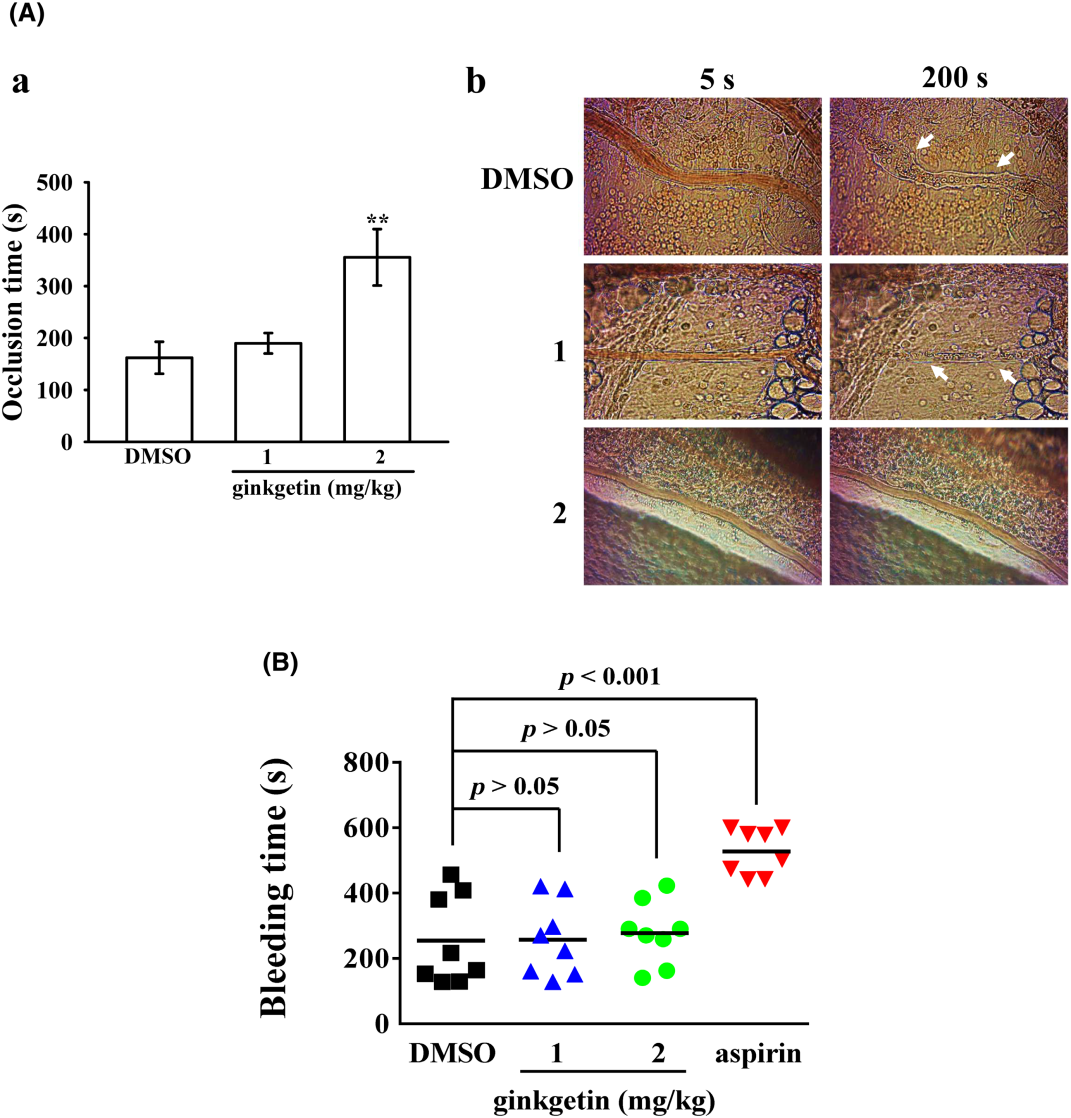
Evaluation of ginkgetin's efficacy in platelet plug occlusion within mesenteric venules and tail bleeding time in mice. In section (A), the study involved the intraperitoneal administration of either a solvent control (0.1% DMSO) or ginkgetin (1 and 2 mg/kg) to mice. (a) Subsequently, mesenteric venules were exposed to fluorescein irradiation to induce microthrombus formation, with the occlusion time being the parameter of interest. (b) Additionally, microscopic images were captured at intervals of 5 and 200 s following irradiation, and the presence of platelet plug formation was identified by white arrows, with magnification at 400×. Data are presented as the mean ± standard error of the mean (*n* = 8). **p < 0.01 compared with the 0.1% DMSO‐treated mice. In section (B), the bleeding time was determined through the transection of mouse tails, following a 30‐min interval after intraperitoneal administration of either 0.1% DMSO or ginkgetin (1 and 2 mg/kg), as well as 2 mg/kg aspirin as a reference. The data are reported as the mean, based on observations from eight mice.

To assess the in vivo antithrombotic efficacy of GK, we conducted experiments involving the evaluation of fluorescein‐induced platelet plug formation within mesenteric microvessels in mice. In mice pretreated with fluorescein sodium (15 μg/kg), the occlusion time in mesenteric microvessels was approximately 160 s when administered 0.1% DMSO (Figure 7Aa). The occlusion time did not exhibit a significant increase when treated with a 1 mg/kg dose of GK. However, a substantial extension in occlusion time was observed following treatment with 2 mg/kg of GK, in stark contrast to the 0.1% DMSO treatment group (where DMSO led to an occlusion time of 162 ± 31 s, 1 mg/kg GK resulted in 190 ± 20 s and 2 mg/kg GK yielded a notably prolonged occlusion time of 355 ± 54 s; *n* = 8; Figure 7Aa). Subsequent to irradiation, thrombotic platelet plug formation became apparent in mesenteric microvessels at the 200‐s mark, while it was absent at the 5‐s interval in the either 0.1% DMSO‐ or 1 mg/kg GK‐treated groups (Figure 7Ab; indicated by white arrows). By contrast, administration of 2 mg/kg GK effectively prevented the formation of platelet plugs at both 5 and 200 s postirradiation (Figure 7Ab). These findings emphasize the promising antithrombotic potential of GK in vivo. Furthermore, the impact on bleeding time was investigated by performing mouse tail transection 30 min after intraperitoneal administration of GK. The bleeding times were measured as follows: 254 ± 48 s (0.1% DMSO‐treated group; *n* = 8), 258 ± 40 s (1 mg/kg GK‐treated group; *n* = 8), 277 ± 34 s (2 mg/kg GK‐treated group; *n* = 8). In the current investigation, aspirin was also utilized as a comparator to assess bleeding time in conjunction with GK. It was observed that the administration of aspirin at a dosage of 2 mg/kg significantly extended the bleeding time within the same experimental conditions (528 ± 25 s; *n* = 8). In the course of this research, individual monitoring of mice persisted for a 15‐min duration following the cessation of bleeding to meticulously ascertain the occurrence of any potential rebleeding events. The results suggest that GK exhibits antithrombotic activity without affecting bleeding time under effective dose.

## 4 DISCUSSION

GK is a flavonoid compound and particularly noteworthy for its medicinal properties. The findings of this study reveal that GK possesses highly potent antiplatelet activity in humans and animal experiments. The concentrations of 0.5 and 1 μM of GK were sufficient for inhibition of platelet activation stimulated by collagen, which is incredibly low. Although GK obtained from natural sources would be insufficient to achieve the required plasma concentrations that can inhibit in vivo platelet activation; however long‐term intake is ideal for preventing atherothrombotic events. Consequently, GK emerges as a compelling candidate for innovative antithrombotic intervention in human subjects, given its pronounced antiplatelet activity.

We additionally investigated the influence of GK on the glycoprotein (GP) VI receptor, as illustrated in Figure S4. Upon exposure to convulxin (25 ng/mL), a specific GP VI agonist known to induce Syk phosphorylation, GK exhibited no discernible impact on the phosphorylation of Syk. This observation suggests that GK does not selectively target individual receptors, such as the collagen receptor, but instead exerts its effects on a common pathway shared by both collagen and AA. We posit that PLCγ2 may play a role in the antiplatelet activity mediated by GK. The activation of PLCγ2 is notably evident when platelets are stimulated by collagen and AA. By contrast, the response is comparatively weaker by thrombin, and no discernible reaction is observed by U46619. PLC exists in two predominant isoforms in human platelets: PLCβ and PLCγ. Remarkably, both of these isoforms contribute distinctively to the signaling pathways triggered by collagen, AA, thrombin and U46619 during platelet activation. Within the PLCγ family, isoforms 1 and 2 are present, with PLCγ2 prominently participating in the signaling pathways initiated by collagen and AA.^24^, ^25^ Collagen is a major component of the extracellular matrix and is exposed upon vascular injury. When collagen binds to specific receptors (i.e. GPVI), it triggers platelet activation through PLCγ2‐dependent pathways.^26^ When platelets are activated by various stimuli, such as collagen, phospholipase enzymes, including phospholipase A_2_ (PLA_2_), are activated. These enzymes cleave AA from the phospholipids in the cell membrane. Once released, AA can be further metabolized by COX to generate prostaglandins, which plays a crucial role in platelet activation. In addition, we also observed that aspirin at a concentration of 100 μM exerts no influence on AA‐triggered PLCγ2 phosphorylation. This suggests that the phosphorylation of PLCγ2 stimulated by AA occurs independently of TxA_2_ generation (Figure S5). Notably, Yeung et al.^25^ have previously proposed that AA could influence PLCγ2 phosphorylation by activating 12‐lipoxygenase rather than through TxA_2_ synthesis in platelets. Therefore, AA and PLCγ2 are interconnected components of platelet signaling pathways, with PLCγ2 playing a central role in the initiation of signaling events, including the generation of second messengers, while AA contributes to downstream processes that enhance platelet activation.^25^ The activity of PLCγ2 is intricately connected to the generation of IP_3_, a crucial second messenger in platelet signaling.^19^ In our present study, we observed a reduction in IP_3_ levels during collagen‐induced platelet activation after treatment with GK (Figure S3). Additionally, we examine TxA_2_ formation stimulated by collagen and AA in human platelets and GK markedly reduced TxB_2_ formation (Figure 2D), indicating that GK does not specifically target individual receptors rather than on COX‐1. Upon activation of Gα_q_‐protein‐coupled receptors (GPCR), Gα_q_ dissociates from the receptor, binds and activates PLCβ isoenzymes, which is crucial for platelet aggregation in response to most GPCR agonist stimulation (i.e. thrombin, ADP and TxA_2_).^27^ However, GK has no effect on PLCβ phosphorylation. Hence, it may be explained why GK exhibited remarkable potency in inhibiting platelet aggregation stimulated by collagen and AA, but not by thrombin or U46619. In the present study, GK effectively suppressed the activation of PLCγ2/PKC activation triggered by collagen stimulation. Intriguingly, GK did not exert a direct influence on PKC activation, as evidenced by the unaltered platelet aggregation response induced by PDBu (Figure 3E). This particular observation suggests that the inhibition of PLCγ2 downstream pathways may constitute a pivotal mechanism through which GK exerts its inhibitory effects on platelet activation.

**FIGURE 8:**
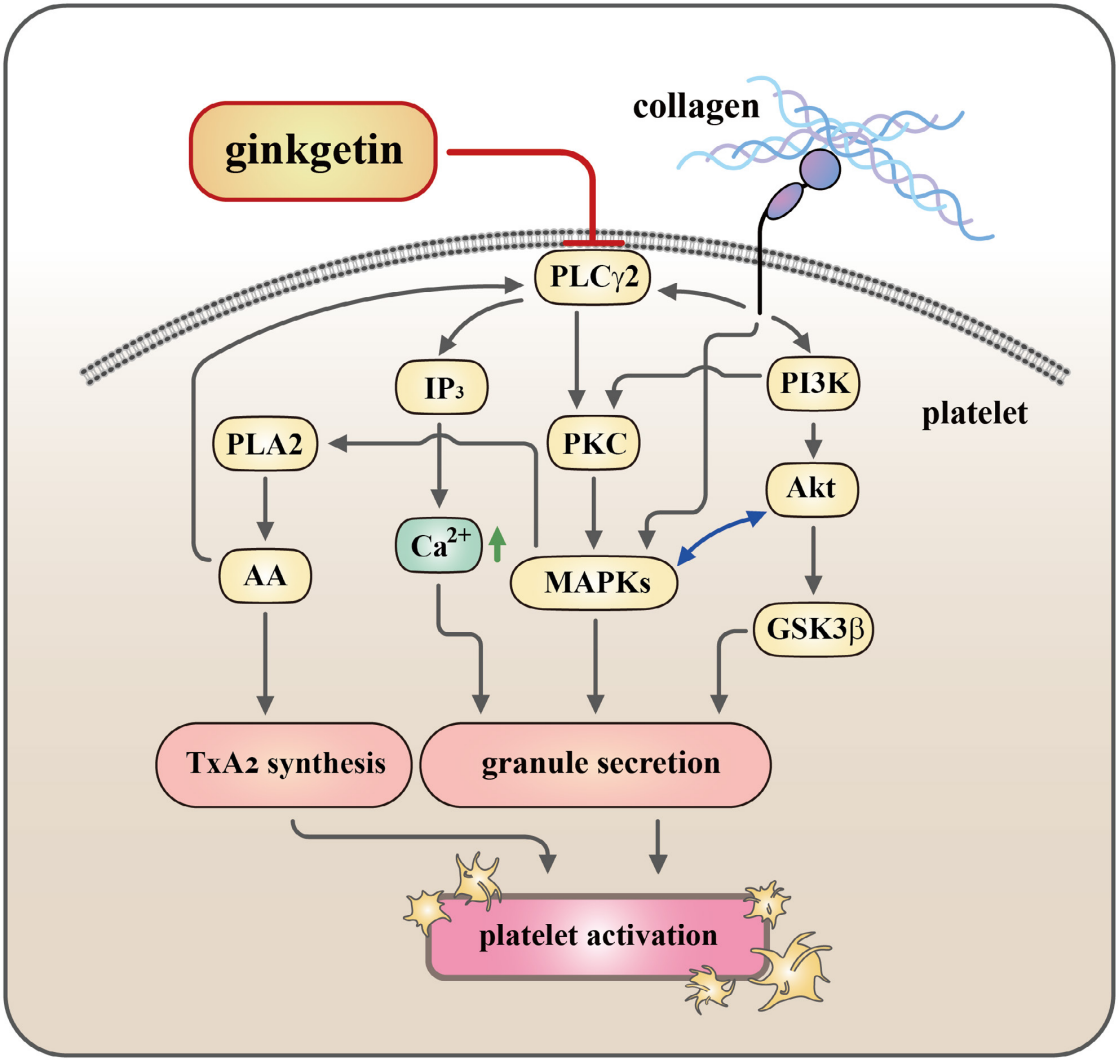
A theoretical framework elucidates the intricate mechanisms that underlie ginkgetin's inhibitory impact on human platelet activation. Ginkgetin exerts its inhibitory effects by targeting key signaling cascades, notably PLCγ2/PKC, PI3K‐Akt‐GSK3β and MAPKs. Subsequently, it orchestrates a meticulous control of intracellular calcium mobilization ([Ca^2+^]i), culminating in the ultimate suppression of platelet aggregation.

The activation of platelets involves intricate signaling pathways, and one of the pivotal contributors to this process is PI3K. PI3K plays a crucial role downstream of various platelet receptors, such as GPVI, which regulates the activation of PLCγ2 and facilitates Ca^2+^ mobilization.^28^ Akt, a major and widely influential effector of PI3K, mice lacking Akt display impaired platelet aggregation and stable adhesion under flow conditions.^29^ Consequently, PI3K–mediated Akt activation, emerges as a promising target for the development of antithrombotic medications. In platelets, PI3K/Akt and MAPKs are mutually activated, with PKC serving as MAPK upstream regulator (Figure 8).^30^ On the contrary, whether the downstream signaling of Akt is involved in platelet activation remains unknown; several candidates such as GSK3 (α and β isoforms) have been identified and expressed in platelets, and GSK3β is the most abundant protein.^31^ Notably, mice with platelet‐specific PI3K deficiency exhibit arterial thrombus instability under conditions of high shear stress due to impaired Akt/GSK3 activation within the developing thrombus.^21^ Nonetheless, the specific mechanisms through which GSK3 regulates platelet activation remain enigmatic. Therefore, the identification of GSK3's substrates within platelets could hold the key to identifying promising targets for the development of novel antithrombotic drugs. Overall, the PI3K/Akt/GSK3β signaling cascade plays a key role in platelet activation and thrombus growth and stability under high shear stress in vivo (Figure 8).

MAPK cascades are vital signaling pathways that intricately control various cellular processes, including proliferation, differentiation and apoptosis. Research involving MAPK‐specific inhibitors and knockout mice has compellingly demonstrated the presence of ERK1/2, JNK1/2 and p38 MAPK in platelet activation.^32^ The specific roles of JNK1/2 and ERK1/2 in platelet activation remain elusive, with intriguing hints suggesting they may act as suppressors of integrin α_IIb_β_3_ activation.^33^ Additionally, the activation of ERK1/2 and JNK1/2 is essential for collagen‐induced platelet aggregation.^34^ Cytosolic PLA_2_ plays a critical role by facilitating the release of AA to generate TxA_2_, a key substrate driving p38 MAPK activation in response to platelet agonists (Figure 8).^34^ Our study revealed that GK significantly inhibits ERK1/2, JNK1/2 and p38 MAPK activation. Furthermore, GK (0.5 and 1 μM) inhibited cPLA_2_ phosphorylation stimulated by collagen (Figure S6), indicating that GK could block TxA_2_ synthesis by regulating cPLA_2_ phosphorylation. This may explain the heightened effectiveness of GK in inhibiting platelet activation induced by collagen or AA.

Raising intracellular cyclic AMP or cyclic GMP levels within platelets activates their respective protein kinase A and protein kinase G pathways. These kinases, in turn, exert their influence by phosphorylating intracellular protein targets, such as VASP, which plays a pivotal role in inhibiting platelet adhesion and aggregation.^35^ Elevated cyclic nucleotide levels have the dual effect of diminishing calcium flux from the dense tubular system and concurrently reducing the activity of cell membrane‐bound calcium transporters. This reduction in calcium levels serves to dampen the activation of PLC/PKC signaling pathways. In our current investigation, it is notable that neither SQ22536 nor ODQ significantly reversed the antiplatelet effects induced by GK. Intriguingly, GK did not seem to affect the phosphorylation status of VASP at both Ser^157^ and Ser^239^. Furthermore, GK did not influence the increasing intracellular levels of cyclic AMP or cyclic GMP in human platelets. These findings emphasize that GK operates through mechanisms that are distinct from the cyclic nucleotide‐VASP axis.

In the pursuit of understanding the therapeutic potential of test compounds against vascular thrombosis, the utilization of animal models holds paramount importance. Among these models, the mouse model stands out as particularly advantageous due to its technical simplicity, expeditious execution and high reproducibility. In a microvascular thrombosis study,^16^ mesenteric venules were continuously exposed to fluorescein irradiation throughout the experimental duration, inducing substantial injury to the endothelial cells. While the impact is not solely on platelets, the modulation of thrombus formation involves multiple factors, including coagulation factors, platelets and blood vessels. This animal model mainly focuses on the initiation of platelet activation induced by endothelial cell damage through irradiation of the mesenteric venules with filtered light. This results in aggregates consisting almost exclusively of platelets with pseudopod formation and a degranulated appearance.^36^ Remarkably, treatment with GK at a dose of 2 mg/kg resulted in a significant extension of occlusion time. Aspirin stands as one of the most widely utilized antiplatelet therapies for both primary and secondary prevention of CVDs, it is not without its drawbacks, particularly the unwanted prolongation of bleeding time. In this study, we observed that the bleeding time of GK‐treated mice remained unchanged and was same as the bleeding time observed in the control mice. This finding suggests that GK may indeed be considered a promising natural compound for the treatment of thromboembolic disorders.

In conclusion, the promotion of healthy dietary and lifestyle habits stands as a pivotal strategy for the modifiable prevention of CVDs at their earliest stages. Our investigations unveiled that GK exerts a potent inhibitory effect on platelet activation, achieved through the inhibition of the PLCγ2–PKC cascade. Consequently, this leads to a subsequent suppression of the PI3K‐Akt and MAPK signaling pathways. This multifaceted action culminates in the reduction of ([Ca^2+^]i) and a consequential inhibition of platelet aggregation (Figure 8). From this study underscore the potential therapeutic and prophylactic applications of GK in the CVDs.

## AUTHOR CONTRIBUTIONS

**Chih‐Wei Hsia:** Formal analysis (equal); investigation (lead); methodology (lead); validation (equal); writing – original draft (lead). **Lan‐Hsin Shu:** Formal analysis (equal); investigation (equal); validation (equal). **Ai‐Wei Lee:** Formal analysis (lead); methodology (lead); validation (equal); writing – review and editing (lead). **Oanh‐Thi Tran:** Formal analysis (equal); investigation (equal); validation (equal). **Chih‐hao Yang:** Data curation (lead); formal analysis (equal); investigation (equal). **Ting‐Lin Yen:** Formal analysis (equal); investigation (equal); visualization (lead). **Wei‐Chieh Huang:** Formal analysis (equal); investigation (supporting). **Chih‐Hsuan Hsia:** Formal analysis (supporting); investigation (equal). **Thanasekaran Jayakumar:** Formal analysis (equal); investigation (supporting). **Kuan‐Rau Chiou:** Conceptualization (equal); resources (lead); supervision (equal); writing – review and editing (equal). **Joen‐Rong Sheu:** Conceptualization (lead); project administration (lead); resources (equal); supervision (equal); writing – original draft (lead).

## CONFLICT OF INTEREST STATEMENT

The authors have no conflicts of interest to declare.

## Supporting information


Figures S1–S6

## Data Availability

All data generated or analyzed during this study are included in the manuscript.
