# Supplementary material for: Ginkgetin effectively mitigates collagen and AA‐induced platelet activation via PLCγ2 but not cyclic nucleotide‐dependent pathway in human
Source: J Cell Mol Med. 2024 Feb 9;28(4):e18139. doi: 10.1111/jcmm.18139 (PMC10853947; doi:10.1111/jcmm.18139)
Supplement: Supplementary file 1 — Figures S1–S6 [file JCMM-28-e18139-s001.pdf]

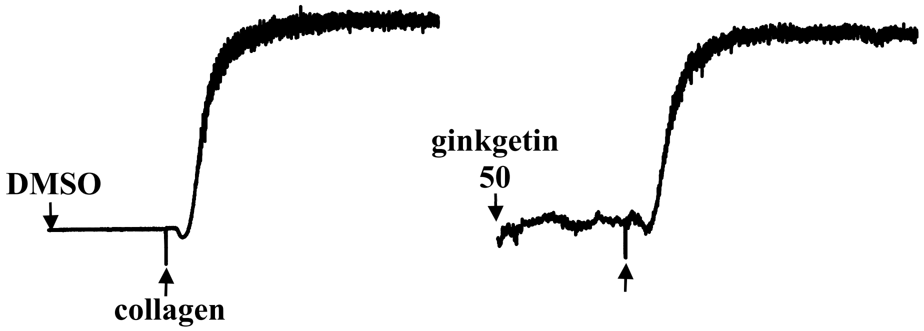

FIGURE S1. The effect of ginkgetin on cytotoxicity in human platelets was investigated. Washed platelets were preincubated with the solvent control (0.1% DMSO) or ginkgetin (50 μM) for 10 min and subsequently washed two times with Tyrode's solution. Collagen (1 μg/mL) was then added to trigger platelet aggregation. The profiles are representative of three independent experiments.

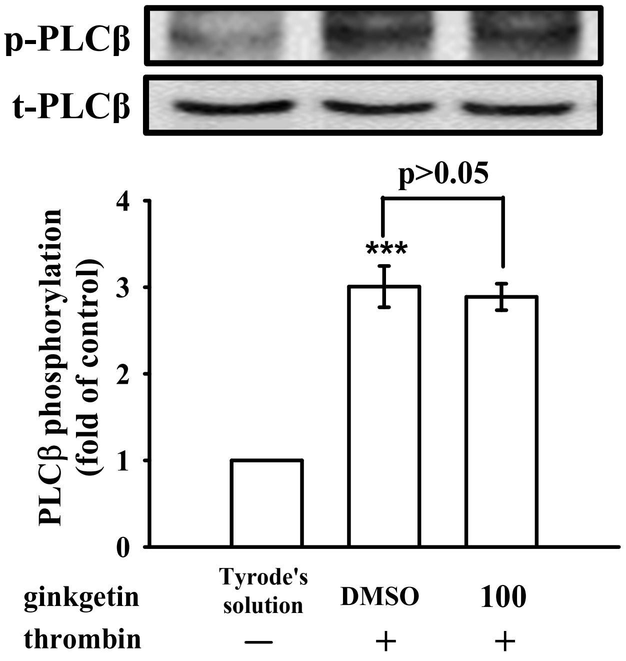

FIGURE S2. Effect of ginkgetin on PLCβ phosphorylation in platelets. Washed platelets were subjected to preincubation with either 0.1% DMSO or ginkgetin (100 μM) and subsequently exposed to thrombin (0.02 U/ml) to induce PLCβ phosphorylation. Data are presented as the mean ± standard error of the mean (n = 3). Significant differences are indicated by $^{***}p < 0.001$ in comparison to resting platelets exposed to Tyrode's solution.

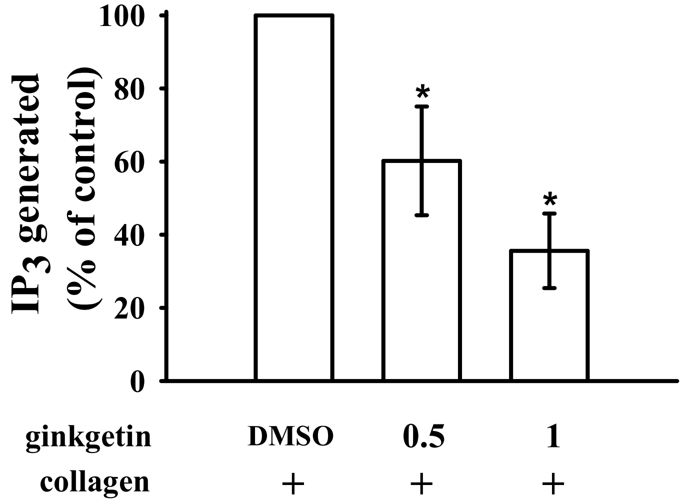

FIGURE S3. The impact of ginkgetin on inositol 1,4,5-trisphosphate (IP_3) generation in human platelets. Washed platelets (3.6×10^8 cells/mL) were subjected to preincubation with either 0.1% DMSO or ginkgetin (0.5 and 1 μM), followed by the addition of collagen (1 μg/mL) to detect inositol 1,4,5-trisphosphate (IP_3) level by ELISA kit (Elabscience, Wuhan, China). The data presented as the mean ± standard error of the mean (n = 3). Significant differences are indicated by $^*p < 0.05$ in comparison to the 0.1% DMSO-treated group.

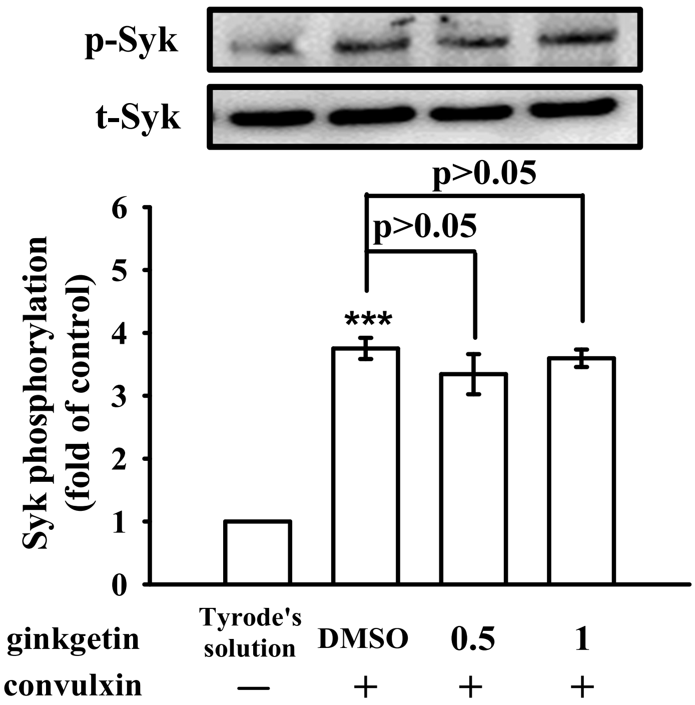

FIGURE S4. Regulatory influence of ginkgetin on the activation of Syk phosphorylation in convulxin-induced platelet activation. Washed platelets (3.6×10^8 cells/mL) were preincubated with either 0.1% DMSO or ginkgetin (0.5 and 1 μM) and subsequently stimulated by convulxin (25 ng/mL) to induce Syk phosphorylation. Data are presented as the mean ± standard error of the mean (n = 3). Significant differences are indicated by $^{***}p < 0.001$ in comparison to resting platelets exposed to Tyrode's solution.

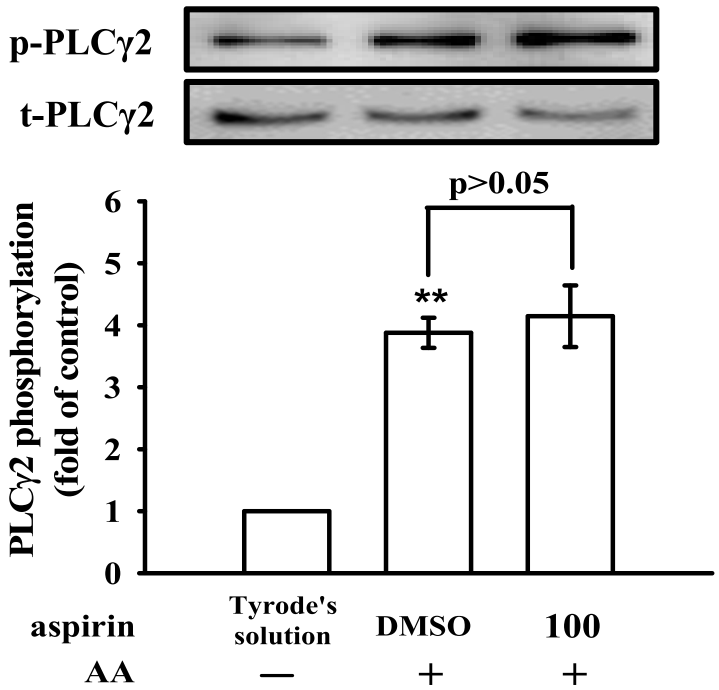

FIGURE S5. Effect of aspirin on PLCγ2 phosphorylation stimulated by arachidonic acid in platelets. Washed platelets were preincubated with 0.1% DMSO or aspirin (100 μM) and subsequently stimulated by arachidonic acid (AA; 60 μM) to induce PLCγ2 phosphorylation. Data are presented as the mean ± standard error of the mean (n = 3). Significant differences are indicated by $^{**}p < 0.01$ in comparison to resting platelets exposed to Tyrode's solution.

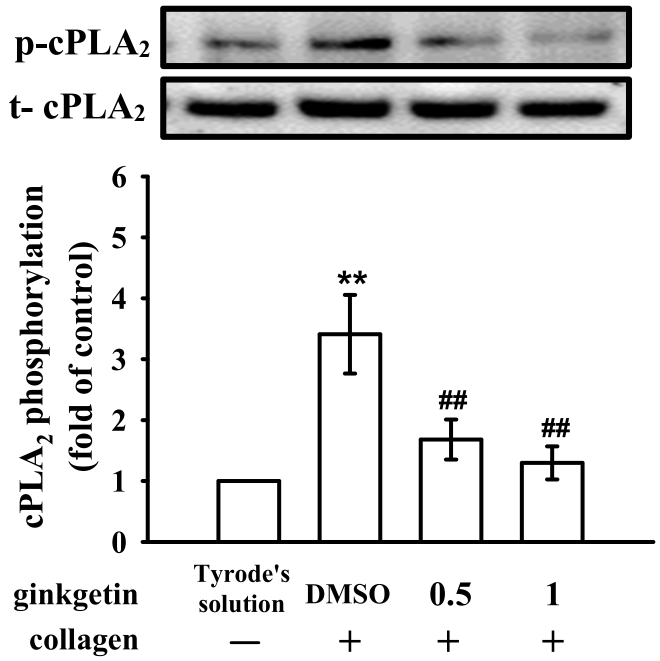

FIGURE S6. Effect of ginkgetin on $cPLA_2$ phosphorylation in collagen-induced platelet activation. Washed platelets (3.6×10^8 cells/mL) were preincubated with either 0.1% DMSO or ginkgetin (0.5 and 1 μM) and subsequently stimulated by collagen (1 μg/mL) to induce $cPLA_2$ phosphorylation. Data are presented as the mean ± standard error of the mean (n = 3). Significant differences are indicated by $^{**}p < 0.01$ in comparison to resting platelets exposed to Tyrode's solution and $^{\#\#}p < 0.01$ in comparison to the 0.1% DMSO group.
